# The Long-Term Results of Covered Endovascular Aortic Bifurcation Repair in Complex Aortoiliac Disease: A Two-Year Follow-Up

**DOI:** 10.3390/jcm13195684

**Published:** 2024-09-24

**Authors:** Nur Dikmen, Evren Ozcinar, Fatma Akça, Emre Sen, Ali Fuat Karacuha, Ahmet Kayan, Levent Yazicioglu

**Affiliations:** 1 Faculty of Medicine, Cardiovascular Surgery Department, Ankara University, 06100 Ankara, Türkiye; nurdikmen@yahoo.com (N.D.); dremresen@outlook.com (E.S.); leventyazicioglu@hotmail.com (L.Y.); 2 Kirikkale High Specialization Hospital, 71300 Kırıkkale, Türkiye; akcaafatma@gmail.com (F.A.); dr.ahmet.kayan@gmail.com (A.K.); 3 Trabzon Kanuni Education and Research Hospital, 61250 Trabzon, Türkiye; alifuatkaracuha@hotmail.com

**Keywords:** aortoiliac occlusive disease, aortic bifurcation, CERAB procedure, endovascular

## Abstract

**Background:** We aimed to investigate the two-year outcomes of covered endovascular reconstruction (CERAB) of the aortic bifurcation in patients with complex aortoiliac occlusive dis ease. **Methods:** This study was prospectively initiated, with data retrospectively collected from 40 patients categorized as TASC II B, C, and D based on computed tomography angiography (CTA) findings. All patients underwent the CERAB procedure. We assessed the procedural outcomes, including clinical and symptomatic improvements, as well as patency rates over a two-year follow-up period. **Results:** A total of 40 patients (33 males and 7 females) with aorto-occlusive disease were treated using the CERAB procedure and included in this observational study. The technical success rate was 100% across all procedures. At 36 months, the overall primary patency, assisted primary patency, and secondary patency rates were 85%, 90%, and 92.5%, respectively. **Conclusions:** The two-year results of this study suggest that CERAB offers patency rates comparable to those reported in other studies for complex aorto-occlusive bifurcation diseases. The procedure showed favorable patency rates, particularly for more advanced TASC II B, C, and D lesions.

## 1. Introduction

Peripheral artery disease (PAD) is a prevalent vascular condition predominantly resulting from atherosclerosis, which accounts for approximately 95% of cases. A significant subset of PAD is aortoiliac occlusive disease (AIOD), typically characterized by symptoms such as claudication and chronic limb-threatening ischemia. However, due to the development of collateral circulation, many patients remain asymptomatic, complicating the accurate assessment of AIOD’s incidence and prevalence. Notably, around 30% of arterial lesions involve the aortoiliac segment, leading to the clinical manifestation of AIOD [1,2,3,4].

In recent years, the management of complex aorto-occlusive cases, particularly in patients at high surgical risk, has gained prominence in the field of vascular intervention. The 2024 European Society of Cardiology (ESC) Guidelines for the management of peripheral arterial and aortic diseases emphasize a multidisciplinary approach to treatment, advocating for endovascular techniques as first-line therapies in appropriate patients [5]. Our clinic has adopted a hybrid endovascular approach over the past two years, integrating femoral endarterectomy and patchplasty when significant calcification extends to the common femoral artery. This strategy aims to ensure optimal recanalization in challenging cases.

Among various endovascular strategies for treating aortoiliac obstructive disease involving the aortic bifurcation, techniques such as kissing stents, low-profile endografts, and the Covered Endovascular Reconstruction of Aortic Bifurcation (CERAB) technique have emerged as important options. The CERAB technique, in particular, has garnered attention for its potential to achieve optimal anatomical and physiological reconstruction. The preference for covered stents in this procedure is supported by evidence from the COBEST trial, which demonstrated the superiority of covered stents over bare stents for TASC II C and D lesions in terms of patency rates and survival benefits. The advantages of covered stents are especially pronounced in larger, more complex lesions, where they may also reduce the incidence of complications during treatment. By completely covering wall thrombus and ulcerated plaques, covered stents help prevent distal embolization, thereby enhancing their clinical utility [6,7].

This study aims to present our experience with the initial 40 CERAB procedures along with the follow-up results over a two-year period. By contributing to the growing body of evidence regarding the efficacy of this technique in managing AIOD, we hope to provide insights that may guide clinical practice and improve patient outcomes [8,9,10].

## 2. Materials and Methods

### 2.1. Study Design 

In this single-center observational study involving both prospective data collection and a retrospective analysis, data were gathered from patients who underwent the CERAB procedure for severe aorto-occlusive disease at our clinic between March 2021 and January 2024. This study was conducted according to the guidelines of the Declaration of Helsinki, and the research ethics board at Ankara University approved this study (date: 28 August 2023; no: 2023/397). Written informed consent was obtained from the patients. Clinical data were gathered in a dedicated, anonymized institutional health care database and analyzed retrospectively.

The procedures were performed at a single center by a uniform surgical team within the Cardiovascular Surgery Department. Patients experiencing acute limb ischemia due to acute aorto-occlusive embolic disease or those with occlusions resulting from dissection were excluded from the study.

Anatomical suitability for the CERAB procedure was evaluated through the analysis of CTA images of the patients. Demographic characteristics, cardiac function based on echocardiogram findings, medical history, and comorbidities were documented, and the Ankle–Brachial Index (ABI) was measured.

**Figure 1:**
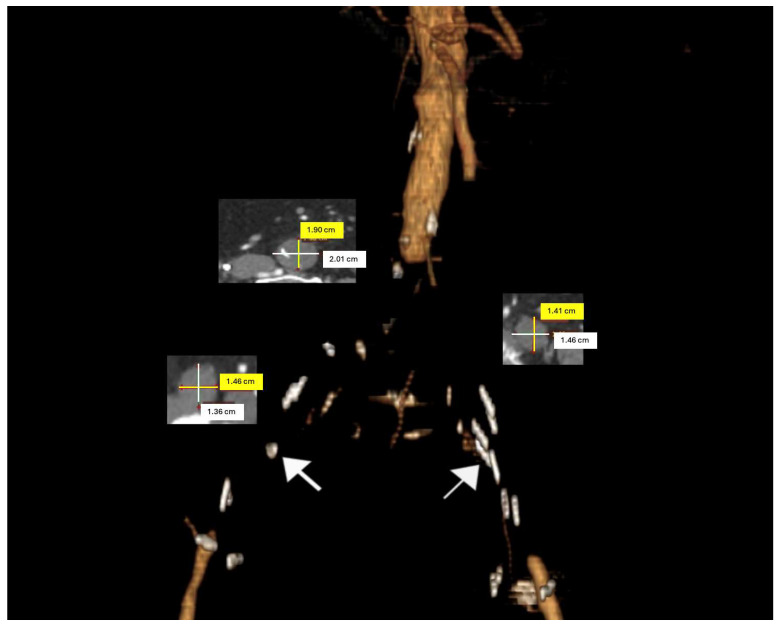
A three-dimensional preoperative model of an aorto-occlusive lesion in an 85-year-old male patient. Axial computed tomography angiography (CTA) sections were utilized to measure the diameters of the distal aorta and common iliac arteries. (The white arrows indicate calcifications on the arterial wall).

Patients were categorized according to TASC II criteria based on their CTA images. (Figure 1). The American Society of Anesthesiologists (ASA) classification was utilized to assess preoperative physical status and operative risks. Clinical presentations were evaluated using the Rutherford classification. In the absence of contraindications, preoperative dual antiplatelet and statin therapy were initiated and maintained throughout the postoperative period.

Postoperative follow-up examinations were conducted within the first week of discharge and at 1, 3, 6, 12, and 24 months. Routine follow-up included assessments of clinical recovery, bilateral ABI measurements, and Doppler ultrasonography (USG). Additionally, CTA control imaging was performed at 6, 12, and 24 months.

The primary outcomes assessed were the patency rates over a 2-year follow-up period. Secondary outcomes included technical success, morbidity and mortality rates, clinical improvement, and limb salvage. Primary patency was defined as the absence of restenosis or occlusion in the treated vessel that would necessitate reintervention. Secondary patency referred to the resolution of occlusions in the stent graft following necessary interventions. Clinical improvement was assessed as an increase of at least 0.10 in ABI and a symptomatic improvement of at least one Rutherford category [5].

### 2.2. Surgical Technique

The technical details of the CERAB procedure have been previously described. The procedure was performed in a hybrid operating room under general anesthesia for patients who had completed preoperative preparations.

Intraoperative ultrasound was used to locate the common femoral arteries, and bilateral oblique surgical cut-downs were made at the inguinal ligaments. After coagulating the skin and subcutaneous tissues with cautery, the deep inguinal fascia was incised to expose the common femoral artery. The perivascular tissues were meticulously dissected, retracted with the right ankle, and secured. Following systemic heparinization (10,000 units), the vessel was palpated to identify a clear zone free of calcifications.

Three parallel 5-0 Prolene sutures were placed along the common femoral artery, and bilateral 9 Fr sheaths were introduced through the central portion of these sutures. A catheter and guide wire (V-18 ControlWire Guidewire, 0.35 inc, Boston Scientific Corporation, Marlborough, MA, USA) were used to cross the occlusive lesion. Subsequently, an aortography was performed to confirm the endoluminal placement of the catheter within the distal aorta.

**Figure 2:**
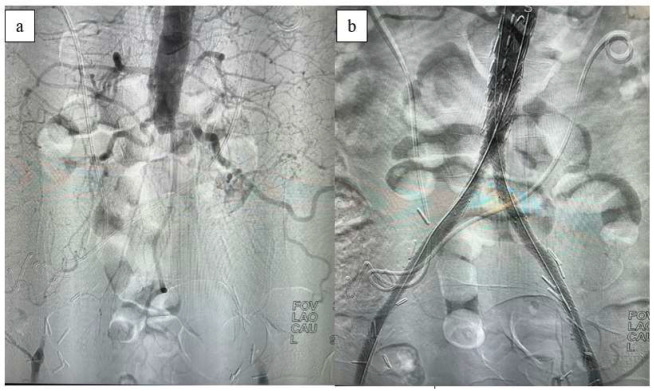
Intraoperative (**a**) and post-CERAB (**b**) aortography.

When the iliac lesion is complete, brachial access is also utilized for aortography. If the brachial artery diameter is adequate, a sheath is placed in the antecubital fossa using the Seldinger technique under ultrasound guidance. For sheaths larger than 6 Fr, access to the brachial artery is achieved via the cut-down technique. The lesion is crossed antegradely through the brachial access, and the guidewire is captured with a snare from the femoral artery to continue the procedure. If the common femoral artery is entirely occluded with no discernible lumen, an alternative approach involves creating retrograde access by initially performing an endarterectomy (Figure 2).

A 12 mm BeGraft Aortic balloon-expandable ePTFE-covered stent (Bentley, InnoMed GmbH, Hechingen, Germany) is expanded in the distal aorta approximately 20 mm above the bifurcation. The proximal two-thirds of the aortic stent is expanded with a larger balloon to allow the distal stent to adopt a funnel shape. Subsequently, bilateral 8 mm covered iliac stents (Bentley Peripheral) are inserted into this funnel-shaped segment, overlapping the aortic stent by one-third of their length. Balloon inflation is performed synchronously to achieve a kissing formation. This technique establishes a tight connection between the iliac stents and the aortic stent, creating a new aortic bifurcation configuration.

Following control angiography, the sheaths are removed from the common femoral arteries, and hemostasis is achieved with purse-string sutures. If the common femoral artery shows inadequate lumen due to extensive calcification, femoral endarterectomy and patchplasty are added to the procedure. After the procedure, patients are extubated, monitored in the intensive care unit for 24 h, and administered low-molecular-weight heparin in addition to dual antiplatelet therapy due to limited mobilization during hospitalization.

### 2.3. Statistical Analysis

Data obtained from this study were analyzed using SPSS Inc., Chicago, IL, USA, version 20. Demographic characteristics and clinical findings were summarized with descriptive statistics. Categorical variables were reported as frequencies, and continuous variables as means ± standard deviations or medians with interquartile ranges (IQRs). The technical success of the procedures and patency rates were expressed as percentages (%). To assess whether there were statistically significant differences in patency rates at two years, a Kaplan–Meier analysis was employed. Differences between groups were evaluated at a significance level of *p* < 0.05.

Additionally, an ANOVA was conducted to evaluate the ABI measurement results from follow-up assessments. For complication rates, the Chi-square test was performed, yielding a Chi-square statistic of 20.11 and a *p*-value of 0.17. This *p*-value exceeds the conventional significance threshold of 0.05, indicating insufficient evidence to suggest a statistically significant difference in complication rates across the different categories. Thus, the observed differences in complication rates may be attributed to random chance.

## 3. Results

During the two-year study period, 40 patients (33 males and 7 females) underwent the CERAB procedure due to aorto-occlusive disease and were included in this observational study. Three patients who additionally underwent the chimney procedure during the study period were excluded, as were fifteen patients who had open surgery with aortobifemoral bypass graft implantation.

The mean age of the study group was 63.08 years (±8.95). Out of the 29 patients diagnosed with coronary artery disease, 12 (41.37%) had an ejection fraction of 40%. The baseline characteristics of the patients are detailed in Table 1.

Chronic kidney disease was present in 18 patients (45%), with 3 of these patients receiving permanent hemodialysis treatment. During the 2-year follow-up period, none of the patients required amputation. The patients were classified according to the Rutherford classification as categories 2 to 4. Based on the review of preoperative CTA imaging, the patients were categorized according to the TASC II score into groups B, C, and D. Among these, 26 patients (65%) were classified as category D, indicating severe disease.

This table summarizes the demographic and clinical characteristics of patients (*n* = 40) who underwent the CERAB procedure. The cohort consisted of 82.5% male and 17.5% female patients with a mean age of 63.08 years (±8.95). The prevalence of comorbidities included diabetes mellitus (57.5%), coronary artery disease (72.5%), hypertension (70%), and chronic kidney disease (45%). Smoking history revealed that 85% of patients were current smokers, while 27.5% were ex smokers for more than 10 years. The ASA scores indicate that the majority (52.5%) were classified as ASA 3. In terms of clinical presentation, the Rutherford classification indicated that 52.5% of patients had class 3 chronic limb ischemia, and the TASC II classification revealed that 65% of lesions were classified as TASC II D. The glomerular filtration rates (GFRs) varied among patients, with 12.5% having a GFR greater than 90. These characteristics provide insights into the population undergoing the CERAB procedure and highlight the complexity of their medical conditions.

Postprocedural outcomes are summarized in Table 2. The CERAB procedure was successfully completed in all patients, achieving a technical success rate of 100%. In many cases, open surgery would have been the only alternative treatment modality. The mean duration of the procedure was 164 min (±38), and the mean hospital stay for patients was 4.07 days (±1.4).

This table presents the postprocedural outcomes for the 40 patients who underwent the CERAB procedure. Technical success was achieved in all cases with a success rate of 100%. The mean amount of contrast agent used during the procedure was 113 mL (±12.8). The average total operation time was 164 min (±38). The patients had a mean length of stay in the intensive care unit (ICU) of 1.3 days (±3.4), and the average length of hospital stay was 4.07 days (±1.4). These outcomes reflect the efficiency and effectiveness of the CERAB procedure in managing aorto-occlusive disease.

There was no 30-day mortality; however, four patients died during the follow-up period. The earliest death, attributed to pulmonary causes, occurred on the 36th day post-procedure. The details of the patients who died due to non-procedural causes are shown in Table 3. One patient required hemodiafiltration for acute renal failure postoperatively and subsequently died after treatment for pneumonia. Another patient was diagnosed with mesenteric ischemia, which prolonged the ICU stay but did not affect mortality. A third patient, who had a low ejection fraction and an implantable cardioverter-defibrillator (ICD), developed decompensated heart failure in the postoperative period and died on the 42nd day.

This table outlines the causes of mortality among patients during the follow-up period following the Covered Endovascular Reconstruction of Aortic Bifurcation (CERAB) procedure. A total of four mortality cases were recorded: one patient died due to pulmonary complications 36 days post-operation, one due to cardiac issues 42 days post-operation, another from malignancy 68 days post-operation, and one patient experienced an unknown cause of death 82 days post-operation. These findings highlight the complexity of patient outcomes in the context of aorto-occlusive disease management.

The main complications reported in our study included access site complications (12.5%), renal dysfunction (10%), and dissection (10%). We used the Chi-square test for the value of complication rates and the test shows a Chi-square statistic of 20.11 and a *p*-value of 0.17. This *p*-value is greater than the typical significance threshold (e.g., 0.05), meaning we do not have enough evidence to suggest a statistically significant difference in complication rates across the different categories. In other words, the observed differences in complication rates could be due to random chance. Access site complications (12.5%) and femoral access site complications (10%) show the highest incidence rates in this dataset. Renal dysfunction and dissections both occurred in 10% of cases, which may indicate areas of potential concern during or after the procedure. Intraoperative complications like massive bleeding, stent dislocation, and arterial rupture occurred at a lower rate (2.5%). (Table 4). Patients who experienced renal dysfunction due to contrast solution were managed with intravenous hydration according to their fluid requirements; however, one of these patients ultimately required permanent hemodialysis.

This table summarizes the complications observed in patients who underwent the Covered Endovascular Reconstruction of Aortic Bifurcation (CERAB) procedure, categorized into overall and intraoperative complications, along with their respective incidence rates.

Intraoperative complications included a rupture of the external iliac artery, which was managed by clamping the proximal area with an aortic balloon and performing an iliofemoral bypass using open surgery.

To access site complications, subcutaneous hematomas at the sheath removal site were managed with pressure dressings. In two patients, superficial infections at the femoral incision sites were successfully treated with antibiotics without the need for additional surgical intervention.

**Figure 3:**
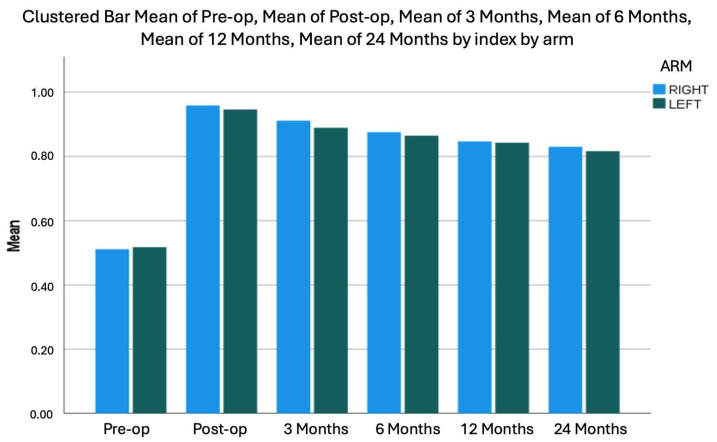
Preoperative and postoperative ABI measurements.

Two cases of arterial rupture and subsequent massive hemorrhage were managed during the procedure with intravenous transfusion and covered stenting. Post-procedure, there was a significant improvement in the ABI, which correlated with the Rutherford classification. The mean ABI, measured bilaterally, significantly improved from approximately 0.5 before the procedure to above 0.8 both immediately after the procedure and throughout the 2-year follow-up period. The one-way ANOVA (repeated measures ANOVA) test result shows an F-statistic of 0.42 and a *p*-value of 0.53. This indicates that there is no statistically significant difference between the mean values for the left and right sides across the different time points (pre-op, post-op, 3 months, etc.) in our dataset for the ABI measurement results from the follow-up assessments. The *p*-value is much higher than the typical significance level (e.g., 0.05), meaning we fail to reject the null hypothesis and conclude that the differences observed are likely due to random variation (Figure 3).

**Figure 4:**
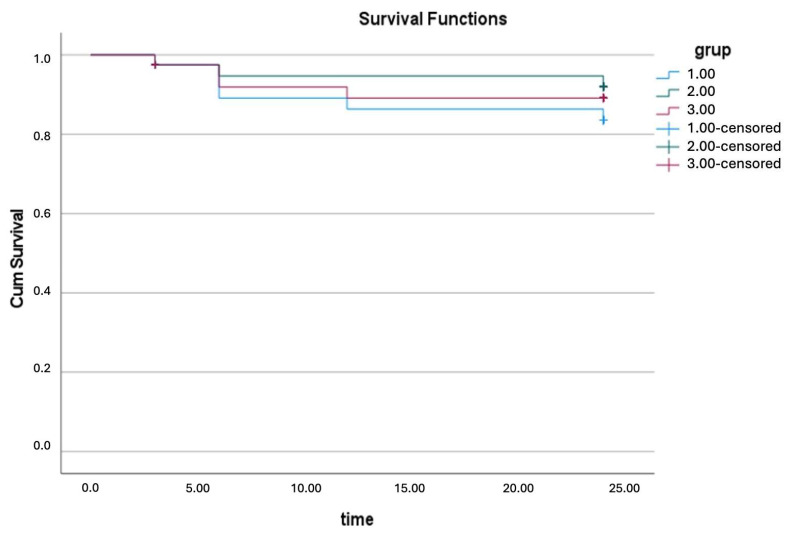
Kaplan–Meier survival analysis showing patency curves of patients treated with CERAB (blue: primary patency; green: secondary patency; red: primary assisted patency). Differences between groups were evaluated at significance level of *p* < 0.05.

The primary patency rate was approximately 98% within the first 6 months but declined to around 85% by the end of the 2-year follow-up period. The secondary patency rate was observed to be 92.5% at the conclusion of the follow-up (Figure 4). During the follow-up, stenosis exceeding 50% developed in four patients. Of these, one patient underwent bilateral iliac percutaneous transluminal angioplasty (PTA), while the others had iliac-femoral PTA and a stent placed in the distal external iliac artery. Additionally, selective thrombolytic therapy was administered to one patient at 16 months due to thrombus formation in the common iliac artery.

## 4. Discussion

The present study underscores the safety and efficacy of the Covered Endovascular Reconstruction of Aortic Bifurcation (CERAB) technique for managing extensive aorto-occlusive lesions at the aortic bifurcation in patients classified as TASC II B, C, and D. Our data reveal a primary patency rate of 85% at the two-year follow-up, which is competitive when compared to the existing literature on both endovascular and surgical interventions. This is particularly noteworthy given the historical preference for open surgical approaches, which have long been considered the gold standard for such complex cases, particularly those classified as TASC II D. Surgical interventions, including aortobifemoral grafts, have reported patency rates reaching as high as 90%. However, these procedures often entail prolonged hospital stays, increased risks of intraoperative complications, and significant postoperative issues, prompting a shift toward less invasive endovascular strategies, particularly for patients at high surgical risk [1,2,11].

The advent of endovascular techniques has revolutionized the management of aortoiliac occlusive disease, with the CERAB technique representing a significant advancement. As introduced by Goverde et al. in 2009, CERAB employs three covered balloon-expandable stents, offering clear advantages over traditional bare stents, particularly for patients with TASC II C and D lesions [1,6].

Our study reinforces this paradigm as the use of covered stents minimizes the risk of distal embolization, a common complication associated with bare stents. This aspect is critical, as covered stents help prevent the potential mismatch area between the stent and the vessel wall that is often seen with the kissing stent technique, which can lead to disturbed blood flow and inadequate perfusion [12,13].

Furthermore, Jebbink et al. demonstrated through a computed tomography analysis that the CERAB technique optimizes flow conditions and reduces radial mismatch, thus enhancing hemodynamic stability in the treated area [14]. This optimization is particularly beneficial in complex cases where significant calcification and anatomical challenges exist. Our clinical results align closely with these findings, providing further validation for the adoption of covered stents in the bifurcation region, particularly in patients with extensive calcific disease.

The integration of intravascular lithotripsy (IVL) into endovascular procedures, as highlighted in the case report by Misuraca et al., has shown promising potential in enhancing procedural outcomes for complex aorto-occlusive lesions. The application of shockwave balloon technology facilitates the treatment of heavily calcified lesions, which are often challenging to manage using conventional techniques. By effectively modifying the calcified plaque structure, IVL promotes improved vessel compliance and enhances the delivery of covered stents, ultimately leading to more favorable deployment and expanded luminal diameter. This innovative approach not only reduces the risk of complications associated with rigid lesions but also contributes to higher primary patency rates. The successful integration of IVL into the Covered Endovascular Reconstruction of Aortic Bifurcation (CERAB) technique underscores the importance of evolving endovascular strategies to optimize outcomes in patients with extensive aorto-iliac occlusive disease [15].

The comparison of patency rates in the literature reveals that our study’s outcomes are consistent with those reported by Taeymans et al., who documented three-year primary, primary assisted, and secondary patency rates of 86%, 91%, and 97%, respectively [16,17]. In contrast, our findings show an 85% primary patency rate at two years, which is comparable to that of the kissing stent technique (79%) and slightly lower than that of the surgical approach (93%). While the primary patency rate is a crucial metric, it is essential to consider secondary patency as well. Our secondary patency rate reached 92.5%, highlighting the effectiveness of a vigilant follow-up and early intervention, particularly within the first 12 months post-procedure, when most reinterventions occurred [7,18].

Additionally, the COBEST trial, the first multicenter study assessing the efficacy of covered stents versus bare-metal stents in treating aortoiliac arterial disease, found a long-term patency rate of 74.7% for covered stents compared to 62.5% for bare-metal stents [9]. These results align with our study’s findings and reinforce the rationale for utilizing covered stents in complex cases, particularly those involving severe lesions classified as TASC II C and D. The CERAB technique, with its anatomical and physiological advantages, as demonstrated by 3D follow-up studies and in vitro analyses, stands to improve patient outcomes when compared to the traditional kissing stent approach [16,17].

Mortality rates are another critical aspect for evaluating surgical interventions for aortoiliac disease. The existing literature indicates a 30-day mortality rate of approximately 20% associated with aortobifemoral and aortobiiliac bypass procedures [18]. In contrast, our study reported no mortality within the first month following the CERAB procedure. By the conclusion of the two-year follow-up, we observed a 25% mortality rate, suggesting a significant improvement compared to traditional open surgical techniques, thereby reinforcing the safety profile of the CERAB method.

In summary, our study indicates that the CERAB technique represents a feasible and effective alternative for the treatment of aortoiliac occlusive disease. With favorable patency rates and a reduced risk of severe complications compared to conventional surgical approaches, CERAB offers a promising avenue for patients, particularly those with high surgical risks. Future research should focus on larger-scale studies to further validate these findings and establish standardized protocols for the management of aortoiliac occlusive disease using endovascular techniques.

## 5. Study Limitations

This study is subject to several limitations that may impact the generalizability of our findings. First, its single-center design restricts the diversity of the patient population, which may not fully represent the broader spectrum of aortoiliac occlusive disease cases encountered in different clinical settings. Additionally, the relatively short follow-up period and small cohort size may limit the robustness of our conclusions regarding the long-term efficacy and safety of the Covered Endovascular Reconstruction of Aortic Bifurcation (CERAB) technique. These factors could introduce biases that affect the assessment of the outcomes and reduce the overall applicability of the results. Despite these limitations, our data provide valuable insights into the feasibility of CERAB for complex aorto-occlusive bifurcation diseases. Future research should focus on addressing these limitations through multicenter randomized trials with larger sample sizes and extended follow-up durations. Such studies are crucial to further validate our findings and enhance their validity across diverse clinical contexts.

## 6. Conclusions

The two-year outcomes of this study indicate that CERAB is an effective treatment for complex aorto-occlusive bifurcation diseases, with patency rates comparable to those reported in the literature for TASC II B, C, and D lesions. Our findings suggest that, with careful patient selection and proficient execution, the CERAB procedure can serve as a viable strategy for managing complex aortoiliac occlusive diseases. Additionally, while the initial results are promising, the necessity for long-term outcome assessments remains critical before CERAB can be widely adopted as a standard treatment modality. Ongoing research is essential to establish definitive guidelines that can optimize patient outcomes and advance the standard of care in the treatment of aortoiliac occlusive disease.

## Figures and Tables

**Table 1:** Baseline characteristics of patients treated with Covered Endovascular Reconstruction of Aortic Bifurcation (CERAB).

	*n* (Total)	%
Male	33	82.5
Female	7	17.5
Age (years) (mean ± SD)	63.08 (±8.95)	
Diabetes mellitus (DM)	23	57.5
Current smoking	34	85
Ex smoker (>10 years)	11	27.5
Ex smoker (<1 year)	11	27.5
Coronary artery disease	29	72.5
Low ejection fraction (<%40)	12	41.37
Hypertension	28	70
Treated with single drug	21	52.5
Treated with two drugs	6	15
Uncontrolled hypertension	1	2.5
Body mass index (BMI)	27.52 ± 8.4	
Hyperlipidemia	31	77.5
Peripheral arterial disease	28	70
Chronic obstructive pulmonary disease	12	30
Chronic kidney disease	18	45
GFR > 90	5	12.5
GFR = 60–90	9	22.5
GFR = 30–60	3	7.5
GFR = 15–30	1	2.5
Carotid disease		
Asymptomatic, but sign of disease	15	37.5
TIA/stroke	1	2.5
ASA score		
1	0	0
2	14	35
3	21	52.5
4	5	12.5
5	0	0
RUTHERFORD classification for chronic limb ischemia		
1	0	0
2	4	10
3	21	52.5
4	10	25
5	5	12.5
6	0	0
TASC II		
A	0	0
B	2	5
C	12	30
D	26	65

Abbreviations: GFR: glomerular filtration rate; TIA: transient ischemic attack; ASA: American Society of Anesthesiologists physical status classification system; TASC II: Trans-Atlantic Inter-Society Consensus (classification of peripheral arterial disease).

**Table 2:** Postprocedural outcomes of patients undergoing Covered Endovascular Reconstruction of Aortic Bifurcation (CERAB).

Postprocedural Outcomes	
Technical success, *n* (%)	40 (100%)
Amount of contrast agent used (mL) (mean ± SD)	113 ± 12.8
Total operation time (minutes) (mean ± SD)	164 ± 38
Length of ICU stay (days) (mean ± SD)	1.3 ± 3.4
Length of hospital stay (days) (mean ± SD)	4.07 ± 1.4

**Table 3:** Causes of mortality during follow-up after CERAB procedure.

Cause of Mortality	Number of Cases	Days Post-Operation
Pulmonary	1	36
Cardiac	1	42
Malignancy	1	68
Unknown	1	82

**Table 4:** Complications following the Covered Endovascular Reconstruction of Aortic Bifurcation (CERAB) procedure.

Type of Complication	Number of Cases (*n*)	Percentage (%)
Overall Complications		
Renal Dysfunction	4	10
Cardiac Complications	1	2.5
Mesenteric Ischemia	1	2.5
Pulmonary Complications	2	5
Access Site Complications	5	12.5
- Femoral Access Site Complications	4	10
- Brachial Access Site Complications	1	2.5
Intraoperative Complications		
- Dissection	4	10
- Massive Bleeding	1	2.5
- Stent Deformation	0	0
- Stent Dislocation	1	2.5
- Distal Embolism	1	2.5
- Thrombus Formation	2	5
- False Aneurysm	0	0
- Groin Hematoma	2	5
- Arterial Rupture	1	2.5

## Data Availability

The original contributions presented in this study are included in this article; further inquiries can be directed to the corresponding authors.

## References

[1] García L.F., Gómez-Rodríguez J.C., Cabrera-Vargas L.F. (2021). Midterm outcomes of the covered endovascular reconstruction of the aortic bifurcation for aortoiliac occlusive disease in a latino american population. Int. J. Surg. Case Rep..

[2] Smith A.H., Beach J.M., Dash S., Rowse J. (2022). Comparison of Aortobifemoral Bypass to Aortoiliac Stenting with Bifurcation Reconstruction for TASC II D Aortoiliac Occlusive Disease. Ann. Vasc. Surg..

[3] Zeller T. (2007). Current state of endovascular treatment of femoro-popliteal artery disease. Vasc. Med..

[4] Bozorghadad S., Scheidt M.J., Patel P.J. (2023). Aortoiliac: Covered, Uncovered, CERAB as It Relates to Peripheral Arterial Disease. Semin. Interv. Radiol..

[5] European Society for Vascular Surgery (ESVS) (2024). Clinical Practice Guidelines on the Management of Asymptomatic Lower Limb Peripheral Arterial Disease and Intermittent Claudication. Eur. J. Vasc. Endovasc. Surg..

[6] Goverde P., Grimme F., Verbruggen P., Reijnen M. (2013). Covered Endovascular Re-construction of the Aortic Bifurcation (CERAB) technique for aortoiliac occlusive disease. J. Cardiovasc. Surg..

[7] Shen C.Y., Qu C.J., Zhang Y.B., Fang J., Teng L.Q., Li J.L. (2023). Midterm Outcomes of Kissing Covered Self-Expanding Stents for Re-construction of Complex Aortoiliac Occlusive Disease. Ann. Vasc. Surg..

[8] Chong A., Mirgolbabaee H., Sun Z., van de Velde L., Jansen S., Doyle B., Versluis M., Reijnen M.M.P.J., Jebbink E.G. (2021). Hemodynamic comparison of stent-grafts for the treatment of aortoiliac occlusive disease. J. Endovasc. Ther..

[9] Mwipatayi B.P., Sharma S., Daneshmand A., Thomas S.D., Vjayan V., Altaf N., Garbowski M., Jackson M., COBEST co-investigators (2016). Durability of the balloon-expandalbe covered versus bare-mwetal stents in the Covered versus Balloon Expandable Stent Trial (COBEST) for the treatmet of the aortoiliac occlusive disease. J. Vasc. Surg..

[10] San Norberto E.M., Revilla Á., Brizuela J.A., Del Blanco I., Flores Á., Taylor J.H. (2024). Combination of BeGraft and Solaris Stent Grafts for the Covered Endovascular Reconstruction of Aortic Bifurcation-BS-CERAB Technique. J. Clin. Med..

[11] Cort B.A., Salemans P.B., Fritschy W.M., Pierie M.E.N., Lind R.C. (2021). Long-term outcome for covered endovascular reconstruction of aortic bifurcation for aortoiliac disease: A single-center experience. J. Endovasc. Ther..

[12] Reijnen M.M. (2020). Update on covered endovascular reconstruction of the aortic bifurcation. Vascular.

[13] Friedman S.A., Holling H.E., Roberts B. (1964). Etiologic factors in aortoiliac and femoropopliteal vascular disease: The Leriche synd-rome. N. Engl. J. Med..

[14] Jebbink E.G., Grimme F.A., Goverde P.C., Oostayen J.A., Slump C.H., Reijnen M.P. (2015). Geometrical consequences of kissing stents and the Covered Endovascular Reconstruction of the Aortic Bifurcation configuration in an in vitro model for endovascular reconstruction of aortic bifurcation. J. Vasc. Surg..

[15] Misuraca L., Buonpane A., Trimarchi G., Picchi A., Filippi F., Biancofiore A., Limbruno U., Rizza A. (2024). Covered Endovascular Reconstruction Of Aortic Bifurcation Facilitated By Intravascular Lithotripsy With Shockwave Balloon: A Case Report. Cureus.

[16] Taeymans K., Jebbink E.G., Holewijn S., Martens J.M., Versluis M., Goverde P.C., Reijnen M.P. (2018). Three-year outcome of the covered endovascular reconstruction of the aortic bifurcation technique for aortoiliac occlusive disease. J. Vasc. Surg..

[17] Kashyap V.S., Pavkov M.L., Bena J.F., Sarac T.P., O’Hara P.J., Lyden S.P., Clair D.G. (2008). The management of severe aortoiliac occlusi-ve disease: Endovascular therapy rivals open reconstruction. J. Vasc. Surg..

[18] Bredahl K., Jensen L.P., Schroeder T.V., Sillesen H., Nielsen H., Eiberg J.P. (2015). Mortality and complications after aortic bifurcated bypass procedures for chronic aortoiliac occlusive disease. J. Vasc. Surg..

